# Supplementary material for: Health-Related Quality of Life and Adherence to Physical Activity and Screen Time Recommendations in Schoolchildren: Longitudinal Cohort Ciao Corona
Source: Int J Public Health. 2023 Jul 19;68:1606033. doi: 10.3389/ijph.2023.1606033 (PMC10394228; doi:10.3389/ijph.2023.1606033)
Supplement: Supplementary file 1 [file DataSheet1.PDF]

Health-related quality of life and adherence to physical activity and screen timerecommendations in schoolchildren: longitudinal cohort Ciao Corona

Supplementary Material

List of Figures

S1 COVID-19 restrictions for schoolchildren in the Canton of Zurich January 2020 - July 2022. Home office for the parents was obligatory (complete) or only recommended by the Federal Council (partial). Schools had to be closed (complete) or the timetables were reduced (partial). Sport and other public facilities were closed (complete) or there were only restrictions of sporting activities and gatherings in public areas (partial). Class camps were either forbidden (complete) or could only be conducted in compliance with a protective concept (partial). Finally, during some periods masks were mandatory in school (complete). 3
S2 Model results for association of HRQOL subscales with adherence to physical activity and screen time recommendations (compared to reference category of not adhering to either recommendation). 4
S3 Model results for association of self-rated health and life satisfaction with adherence to physical activity and screen time recommendations (odds ratio (95% confidence interval)) 5
S4 HRQOL by age at baseline 8

List of Tables

S1 Chronic conditions reported. Lifestyle and health-realted quality of life analysis of Ciao Corona, Switzerland, 2020 - 2022. 2
S2 Percentage of children meeting physical activity (PA) or screen time (ST) recommendations in primary and secondary school, by timepoint (see also Figure 1). 2
S3 Observed HRQOL by adherence to physical activity and screen time recommendations (median [intraquartile range]) 6
S4 Model results for association of HRQOL with adherence to physical activity and screen time recommendations (coefficient (95% confidence interval)) 6
S5 Model results for association of HRQOL physical, emotional and self-esteem subscales with adherence to physical activity and screen time recommendations (coefficient (95% confidence interval)) 6
S6 Model results for association of HRQOL family, friends and school subscales with adherence to physical activity and screen time recommendations (coefficient (95% confidence interval)) 7
S7 Model results for association of self-rated health and life satisfaction with adherence to physical activity and screen time recommendations (odds ratio (95% confidence interval)) 7
S8 Age-stratified model results for association of HRQOL with adherence to physical activity and screen time recommendations (coefficient (95% confidence interval)) . . . 8

Excerpt of questions about physical activity and screen time

1. On a typical weekday (Monday through Friday), how many hours per day does your son/daughter CURRENTLY engage in exercise or other physical activity (that caused sweating or breathing more)? Please count school sports.
2. On a typical weekend day (Saturday, Sunday), how many hours per day does your son/daughter CURRENTLY do sports or other physical activity (that caused sweating or breathing more)?
3. How many hours a day does your son/daughter CURRENTLY spend using electronic devices (e.g. TV, mobile phone, computer, Playstation, Xbox) in their free time on a typical weekday (Monday to Friday)?
4. How many hours a day does your son/daughter CURRENTLY spend using electronic devices (e.g. TV, mobile phone, computer, Playstation, Xbox) in their free time on a typical weekend day (Saturday and Sunday)?

Table S1: Chronic conditions reported. Lifestyle and health-realted quality of life analysis of Ciao Corona, Switzerland, 2020 - 2022.

Variable	N = 1769
Any chronic condition	357 (20%)
Hay fever	253 (14%)
Attention deficit hyperactivity disorder	75 (4.2%)
Asthma	71 (4.0%)
Depression/Anxiety	5 (0.3%)
Epilepsy	4 (0.2%)
Joint disorder, incl. Arthritis	4 (0.2%)
Diabetes mellitus	2 (0.1%)
Inflammatory bowel disease	1 (<0.1%)

Table S2: Percentage of children meeting physical activity (PA) or screen time (ST) recommendations in primary and secondary school, by timepoint (see also Figure 1).

timept	primary_PA	secondary_PA	primary_ST	secondary_ST
baseline	82.9% (934/1126)	77.0% (495/643)	95.3% (1073/1126)	67.5% (434/643)
July 2020	59.4% (669/1126)	51.8% (333/643)	74.4% (838/1126)	28.9% (186/643)
Jan 2021	61.0% (610/1000)	47.9% (276/576)	92.6% (930/1004)	52.7% (305/579)
Mar 2021	74.6% (723/969)	64.7% (346/535)	91.7% (894/975)	52.1% (284/545)
Sept 2021	75.3% (469/623)	63.4% (199/314)	87.0% (535/615)	40.3% (124/308)
July 2022	78.6% (482/613)	65.8% (144/219)	82.2% (493/600)	36.7% (77/210)

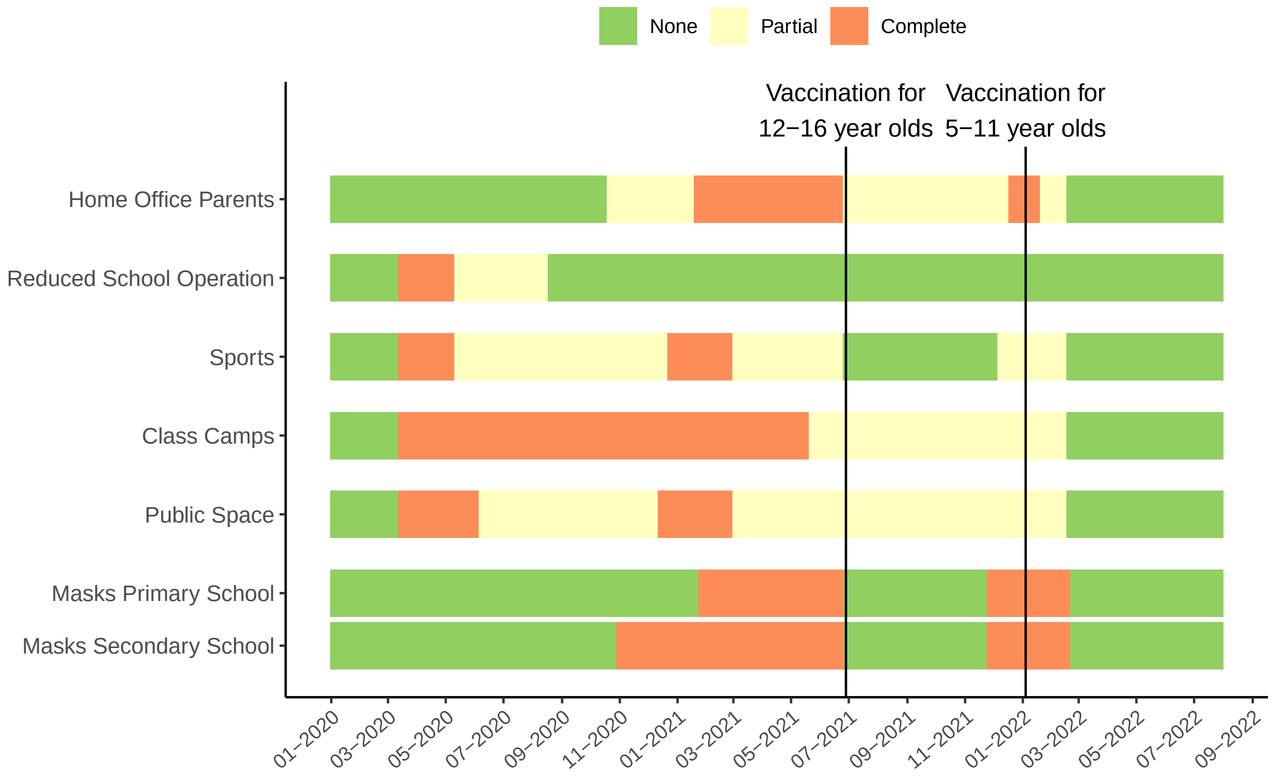

Figure S1: COVID-19 restrictions for schoolchildren in the Canton of Zurich January 2020 - July 2022. Home office for the parents was obligatory (complete) or only recommended by the Federal Council (partial). Schools had to be closed (complete) or the timetables were reduced (partial). Sport and other public facilities were closed (complete) or there were only restrictions of sporting activities and gatherings in public areas (partial). Class camps were either forbidden (complete) or could only be conducted in compliance with a protective concept (partial). Finally, during some periods masks were mandatory in school (complete).

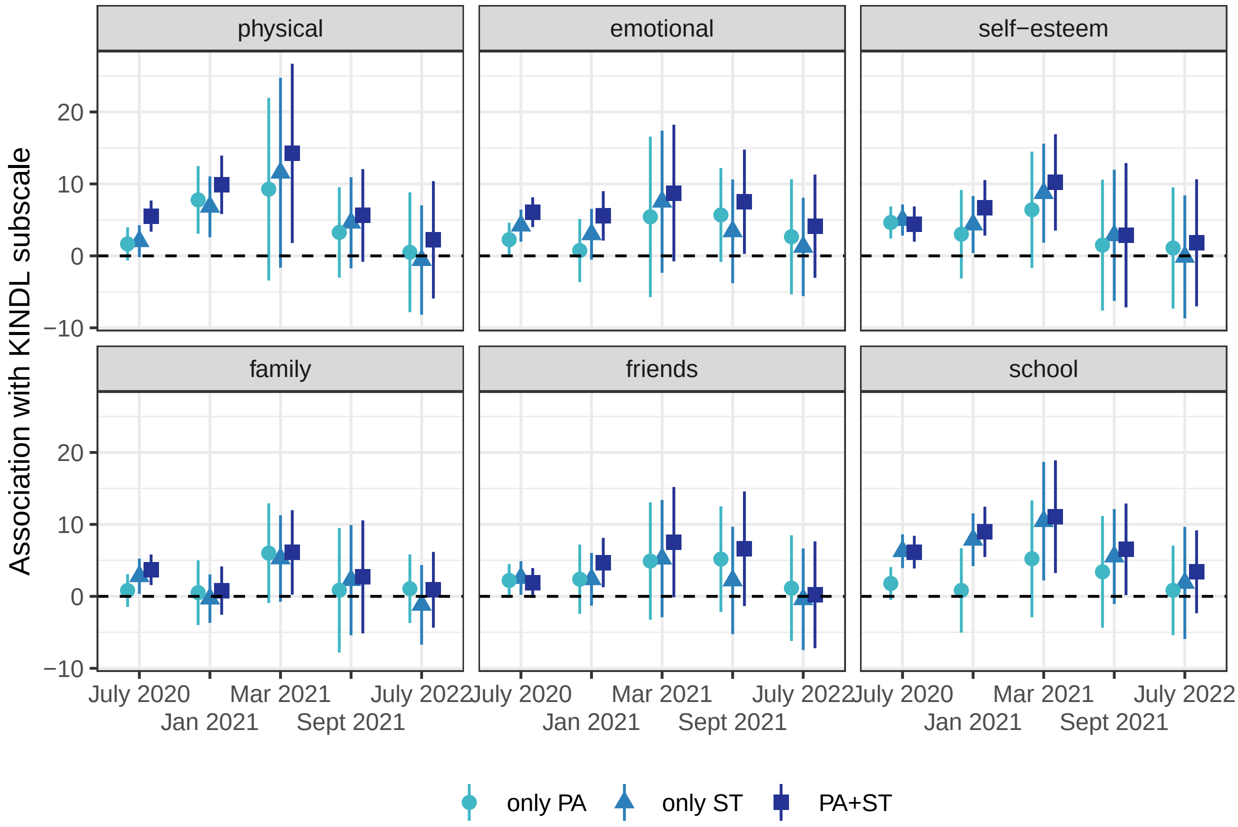

Figure S2: Model results for association of HRQOL subscales with adherence to physical activity and screen time recommendations (compared to reference category of not adhering to either recommendation).

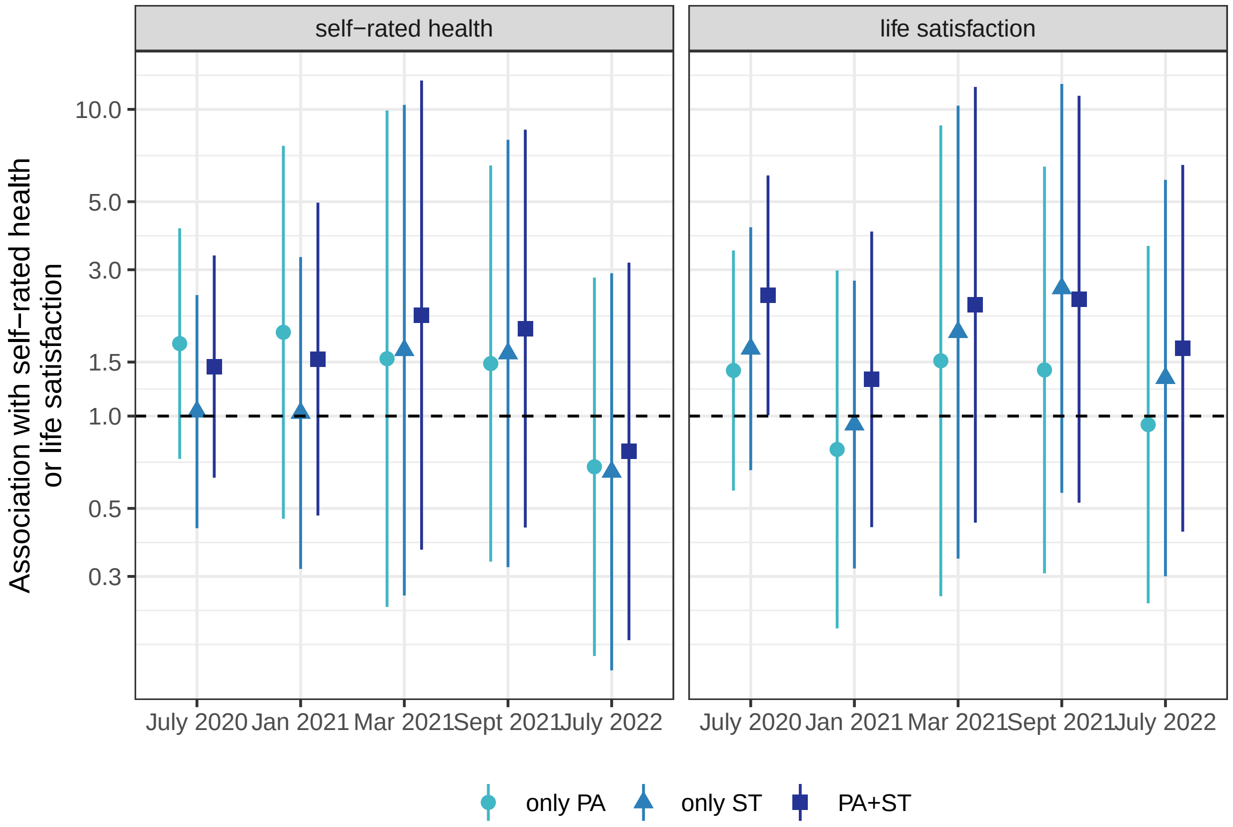

Figure S3: Model results for association of self-rated health and life satisfaction with adherence to physical activity and screen time recommendations (odds ratio (95% confidence interval))

Table S3: Observed HRQOL by adherence to physical activity and screen time recommendations (median [intraquartile range])

timepoint	n	neither	only PA	only ST	PA+ST
July 2020	1769	76.0 [67.7, 82.3]	78.1 [71.9, 84.4]	80.2 [74.0, 85.4]	82.3 [77.1, 87.5]
Jan 2021	1572	71.9 [63.5, 78.1]	72.9 [66.7, 81.2]	77.1 [69.8, 82.3]	80.2 [74.0, 85.4]
Mar 2021	1494	70.8 [62.5, 78.1]	74.0 [66.7, 81.2]	77.1 [71.2, 83.3]	80.2 [72.9, 85.4]
Sept 2021	922	71.9 [63.5, 79.2]	75.0 [67.7, 81.2]	75.0 [68.8, 81.2]	80.2 [74.0, 85.4]
July 2022	809	70.8 [64.6, 78.9]	76.0 [68.9, 82.3]	76.1 [70.8, 81.2]	79.2 [72.9, 85.1]

Table S4: Model results for association of HRQOL with adherence to physical activity and screen time recommendations (coefficient (95% confidence interval))

item	timepoint	neither	only PA	only ST	PA+ST
total	July 2020	(reference)	2.2 (0.4-4.0)	3.8 (1.9-5.7)	4.6 (2.8-6.5)
total	Jan 2021	(reference)	2.0 (-1.8-5.8)	3.5 (0.4-6.7)	5.5 (2.4-8.5)
total	Mar 2021	(reference)	6.2 (-0.5-12.9)	8.1 (1.1-15.2)	9.7 (3.0-16.3)
total	Sept 2021	(reference)	3.2 (-2.3-8.8)	3.4 (-2.0-8.8)	5.2 (-0.7-11.0)
total	July 2022	(reference)	1.7 (-2.3-5.6)	0.5 (-3.4-4.4)	2.4 (-1.3-6.1)

Table S5: Model results for association of HRQOL physical, emotional and self-esteem subscales with adherence to physical activity and screen time recommendations (coefficient (95% confidence interval))

item	timepoint	neither	only PA	only ST	PA+ST
physical	July 2020	(reference)	1.7 (-0.6-4.0)	2.0 (-0.2-4.3)	5.5 (3.4-7.7)
physical	Jan 2021	(reference)	7.8 (3.1-12.5)	6.8 (2.6-11.0)	9.9 (5.8-13.9)
physical	Mar 2021	(reference)	9.3 (-3.4-22.0)	11.6 (-1.6-24.8)	14.2 (1.8-26.7)
physical	Sept 2021	(reference)	3.3 (-3.0-9.5)	4.6 (-1.7-10.9)	5.6 (-0.8-12.1)
physical	July 2022	(reference)	0.5 (-7.8-8.8)	-0.6 (-8.2-7.0)	2.2 (-5.9-10.4)
emotional	July 2020	(reference)	2.3 (-0.1-4.6)	4.2 (2.0-6.4)	6.1 (4.0-8.1)
emotional	Jan 2021	(reference)	0.7 (-3.7-5.1)	3.0 (-0.5-6.5)	5.6 (2.1-9.0)
emotional	Mar 2021	(reference)	5.4 (-5.7-16.6)	7.5 (-2.4-17.4)	8.7 (-0.8-18.2)
emotional	Sept 2021	(reference)	5.7 (-0.8-12.2)	3.4 (-3.8-10.6)	7.5 (0.3-14.8)
emotional	July 2022	(reference)	2.7 (-5.4-10.7)	1.2 (-5.6-8.1)	4.1 (-3.0-11.3)
self-esteem	July 2020	(reference)	4.6 (2.4-6.9)	5.0 (2.8-7.1)	4.4 (2.0-6.9)
self-esteem	Jan 2021	(reference)	3.0 (-3.1-9.2)	4.4 (0.4-8.3)	6.7 (2.8-10.5)
self-esteem	Mar 2021	(reference)	6.4 (-1.7-14.5)	8.7 (1.8-15.6)	10.2 (3.5-16.9)
self-esteem	Sept 2021	(reference)	1.5 (-7.6-10.6)	2.9 (-6.3-12.0)	2.9 (-7.2-12.9)
self-esteem	July 2022	(reference)	1.1 (-7.3-9.5)	-0.1 (-8.7-8.4)	1.8 (-7.0-10.6)

Table S6: Model results for association of HRQOL family, friends and school subscales with adherence to physical activity and screen time recommendations (coefficient (95% confidence interval))

item	timepoint	neither	only PA	only ST	PA+ST
family	July 2020	(reference)	0.8 (-1.5-3.1)	2.8 (0.3-5.3)	3.7 (1.6-5.8)
family	Jan 2021	(reference)	0.5 (-4.0-5.0)	-0.3 (-3.7-3.1)	0.8 (-2.6-4.2)
family	Mar 2021	(reference)	6.0 (-0.9-12.9)	5.3 (-0.7-11.3)	6.1 (0.2-12.0)
family	Sept 2021	(reference)	0.9 (-7.8-9.5)	2.3 (-5.4-9.9)	2.7 (-5.1-10.6)
family	July 2022	(reference)	1.1 (-3.7-5.8)	-1.2 (-6.7-4.4)	0.9 (-4.4-6.2)
friends	July 2020	(reference)	2.2 (-0.1-4.5)	2.6 (0.2-4.9)	1.9 (-0.2-3.9)
friends	Jan 2021	(reference)	2.4 (-2.4-7.2)	2.4 (-1.3-6.0)	4.7 (1.3-8.1)
friends	Mar 2021	(reference)	4.9 (-3.3-13.1)	5.2 (-2.9-13.4)	7.5 (-0.1-15.2)
friends	Sept 2021	(reference)	5.2 (-2.2-12.5)	2.2 (-5.3-9.7)	6.6 (-1.3-14.6)
friends	July 2022	(reference)	1.1 (-6.2-8.5)	-0.4 (-7.5-6.7)	0.2 (-7.2-7.6)
school	July 2020	(reference)	1.8 (-0.5-4.1)	6.3 (3.9-8.6)	6.1 (3.9-8.4)
school	Jan 2021	(reference)	0.8 (-5.0-6.7)	7.9 (4.2-11.5)	9.0 (5.5-12.5)
school	Mar 2021	(reference)	5.2 (-2.9-13.3)	10.4 (2.2-18.7)	11.1 (3.2-18.9)
school	Sept 2021	(reference)	3.4 (-4.4-11.2)	5.5 (-1.1-12.1)	6.5 (0.2-12.9)
school	July 2022	(reference)	0.8 (-5.4-7.1)	1.9 (-5.9-9.7)	3.4 (-2.3-9.2)

Table S7: Model results for association of self-rated health and life satisfaction with adherence to physical activity and screen time recommendations (odds ratio (95% confidence interval))

item	timepoint	neither	only PA	only ST	PA+ST
self-rated health	July 2020	(reference)	1.72 (5.60-5.60)	1.03 (2.81-2.81)	1.45 (4.26-4.26)
self-rated health	Jan 2021	(reference)	1.88 (6.53-6.53)	1.02 (2.78-2.78)	1.53 (4.64-4.64)
self-rated health	Mar 2021	(reference)	1.54 (4.66-4.66)	1.64 (5.15-5.15)	2.13 (8.45-8.45)
self-rated health	Sept 2021	(reference)	1.48 (4.41-4.41)	1.60 (4.95-4.95)	1.93 (6.88-6.88)
self-rated health	July 2022	(reference)	0.68 (1.98-1.98)	0.66 (1.93-1.93)	0.77 (2.15-2.15)
life satisfaction	July 2020	(reference)	1.41 (4.09-4.09)	1.66 (5.25-5.25)	2.48 (11.90-11.90)
life satisfaction	Jan 2021	(reference)	0.78 (2.18-2.18)	0.94 (2.56-2.56)	1.32 (3.73-3.73)
life satisfaction	Mar 2021	(reference)	1.51 (4.55-4.55)	1.88 (6.54-6.54)	2.31 (10.03-10.03)
life satisfaction	Sept 2021	(reference)	1.41 (4.11-4.11)	2.61 (13.58-13.58)	2.40 (11.06-11.06)
life satisfaction	July 2022	(reference)	0.94 (2.55-2.55)	1.33 (3.78-3.78)	1.66 (5.28-5.28)

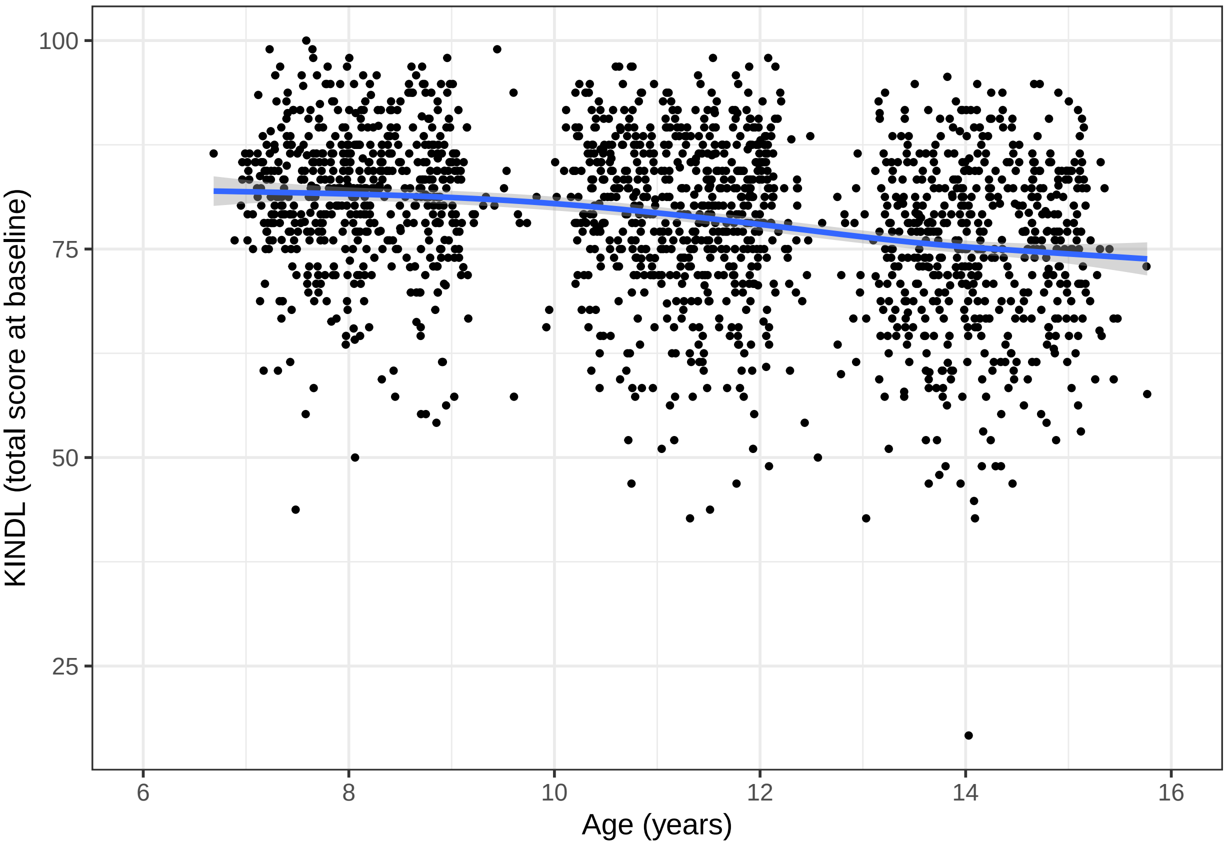

Figure S4: HRQOL by age at baseline

Table S8: Age-stratified model results for association of HRQOL with adherence to physical activity and screen time recommendations (coefficient (95% confidence interval))

grp	item	timepoint	neither	only PA	only ST	PA+ST
primary	total	July 2020	(reference)	2.1 (-0.1-4.2)	3.0 (1.0-5.0)	4.8 (2.8-6.7)
primary	total	Jun 2021	(reference)	2.4 (-3.6-8.4)	4.1 (0.2-8.0)	4.8 (1.0-8.6)
primary	total	Mar 2021	(reference)	0.2 (-8.3-8.7)	3.9 (-4.0-11.8)	5.2 (-2.4-12.8)
primary	total	Sept 2021	(reference)	2.3 (-4.6-9.2)	2.6 (-3.3-8.5)	3.8 (-1.7-9.4)
primary	total	July 2022	(reference)	2.3 (-3.0-7.6)	0.7 (-4.1-5.5)	3.3 (-1.8-8.4)
secondary	total	July 2020	(reference)	1.9 (-0.3-4.1)	4.3 (1.7-6.8)	5.6 (3.2-8.1)
secondary	total	Jun 2021	(reference)	0.1 (-3.0-3.2)	0.6 (-2.2-3.4)	5.0 (2.0-7.9)
secondary	total	Mar 2021	(reference)	14.0 (2.2-25.7)	13.8 (2.3-25.4)	15.3 (3.6-27.0)
secondary	total	Sept 2021	(reference)	2.0 (-3.2-7.2)	1.1 (-4.9-7.0)	4.1 (-2.4-10.6)
secondary	total	July 2022	(reference)	1.3 (-3.0-5.6)	0.6 (-5.2-6.4)	1.1 (-3.8-5.9)
